# Novel Coating Approaches for Polyethylene Biliary Stents to Reduce Microbial Adhesion, Prevent Biofilm Formation, and Prolong Stent Patency

**DOI:** 10.3390/biomedicines13081950

**Published:** 2025-08-09

**Authors:** Laura Wagner, Philipp Stolte, Stephan Heller, Dina Schippers, Dominik Pförringer, Jutta Tübel, Roland M. Schmid, Rainer Burgkart, Jochen Schneider, Andreas Karl-Werner Obermeier

**Affiliations:** 1 TUM School of Medicine and Health, Clinical Department of Internal Medicine II, TUM University Hospital, 81675 Munich, Germany; 2 TUM School of Medicine and Health, Department of Orthopaedics and Sports Orthopaedics, TUM University Hospital, 81675 Munich, Germany; 3 TUM School of Medicine and Health, Department of Surgery, TUM University Hospital, 81675 Munich, Germany; 4 TUM School of Medicine and Health, Department of Trauma Surgery, TUM University Hospital, 81675 Munich, Germany

**Keywords:** biliary stent, occlusion, diamond-like carbon, silver nanoparticles, microbial adherence, biofilm formation

## Abstract

**Background**: Occlusion of plastic biliary stents is a common complication in biliary drainage, often requiring exchange procedures every 2–4 months due to microbial colonization and sludge formation. This study aimed to evaluate diamond-like carbon (DLC) coatings, with and without silver nanoparticle additives, for preventing stent occlusion. **Methods**: Polyethylene (PE) stents were coated with DLC using PlasmaImpax for DLC-1 and pulsed laser deposition for DLC-2. Silver ions (Ag) were incorporated into the DLC-2 coatings. To simulate in vivo conditions, a co-culture of *Enterococcus faecalis (E. faecalis)*, *Escherichia coli (E. coli),* and *Candida albicans (C. albicans)* was used for microbial colonization. Standardized human bile simulated physiological conditions. Adhesion tests, weight measurements, and scanning electron microscopy (SEM) quantified bacterial adherence to stents. **Results**: DLC-1 coatings demonstrated higher bacterial growth than uncoated PE stents with *E. faecalis* (adhesion assay difference: 0.6 log [*p* = 0.19] and 0.1 log [*p* = 0.75] in rounds 1 and 2, respectively). In the bile incubation model, DLC-1 did not significantly reduce bacterial counts at 5 days (0.4 log [*p* = 0.06]) or 14 days (0.2 log [*p* = 0.44]). DLC-2 showed no significant reduction either. DLC-2-Ag significantly reduced bacterial adhesion (5 days: −0.3 log [*p* = 0.00]; 14 days: −0.4 log [*p* = 0.16]) and exhibited inhibition zones against *E. faecalis* (2.3 mm), *E. coli* (2.1 mm), and *C. albicans* (0.6 mm). SEM revealed cracks and flaking in the coating. **Conclusions**: DLC coatings alone did not prevent microbial adhesion. Tendencies of anti-adhesive properties were seen with Ag-doped DLC coatings, which were attributed to the antibacterial effects of Ag. Optimization of the DLC-coating process is needed to improve stent performance. Future studies with larger samples sizes are needed to confirm the observed trends.

## 1. Introduction

In cases of biliary obstruction, bile flow can be restored through endoscopic stent insertion via endoscopic retrograde cholangiography (ERC). Both plastic and metal stents are available, with selection depending on the etiology, length, and location of the obstruction [1]. A common complication of plastic biliary stents is occlusion [2], which occurs earlier than with metal stents due to their smaller diameters. Stent technology limits the diameter of lumens that can be applied, particularly for plastic stents, as they must pass through the endoscope. The median patency of plastic stents is 70–126 days, and the risk of stent occlusion increases significantly after 3 months [2,3,4]. This common complication usually requires a stent replacement every 2–4 months [5]. Hence, plastic stents are changed approximately every 3 months to avoid stent occlusion and associated complications, such as cholangitis [6,7]. The primary cause of stent occlusion is sludge accumulation, driven by microbial colonization and subsequent biofilm formation on the stent surface [8,9]. The composition of microbial colonization changes over time. Initially, aerobic gram-positive bacteria and *Candida species* (spp.) are present. After more than 60 days, aerobic Gram-negative and Gram-positive bacteria, along with anaerobes, become dominant. The most frequently identified microorganisms on biliary stents are *Enterococcus* spp. and *Enterobacteriaceae*, followed by *Candida* spp. [2,3]. Several strategies have been explored to prolong stent patency. In addition to antimicrobial coatings [10], anti-adhesive coatings have emerged as promising alternatives. These coatings modify the stent surface by altering its physicochemical properties to prevent microbial adhesion [11].

A promising strategy to address this challenge involves the application of diamond-like carbon (DLC) coatings doped with antimicrobial elements, such as silver (Ag) or copper (Cu). DLC coatings have also been applied to metallic cardiac stents, where they demonstrated favorable hemocompatibility and reduced clot formation, supporting their clinical relevance in other biomedical fields [12]. Such coatings have demonstrated beneficial properties, including surface smoothness, chemical inertness, biocompatibility, and antimicrobial activity [13,14,15]. Harrasser et al. [16,17] investigated Ag- and Cu-DLC coatings on orthopedic-grade high-density polyethylene (HDPE) and reported significant bactericidal effects, particularly for Ag. Similarly, Gorzelanny et al. [18] showed that comparable Ag concentrations can be both antimicrobial and cytocompatible, supporting a potential therapeutic window for Ag-DLC coatings on soft biomaterials. However, their study used ion implantation techniques at elevated process temperatures—conditions that are not compatible with the thermally sensitive materials used in polymeric biliary stents. In addition, their work was performed on orthopedic implant substrates, under conditions that do not reflect the complex composition of human bile or its associated microbiota. Birkett et al. [19] and Vishwakarma et al. [20] recently reviewed multifunctional DLC coatings and emphasized their potential for various medical applications. However, most of the published studies focus on orthopedic or cardiovascular environments. While polymeric stents with DLC coatings have been studied in urological settings, their efficacy in bile-exposed environments remains unexplored [21]. Furthermore, no previous study has examined Ag-DLC coatings in the context of thermally labile polymeric biliary stents under biliary-specific conditions—such as bile exposure and mixed-species biofilm formation involving Gram-positive, Gram-negative bacteria, and fungal organisms (e.g., *Escherichia coli (E. coli)*, *Enterococcus faecalis (E. faecalis)*, and *Candida albicans (C. albicans)*).

To address this gap, our study adapts established DLC coating processes (PlasmaImpax and pulsed laser deposition (PLD)) to medical-grade polyethylene (PE) stents, using low-temperature settings and other process parameters compatible with the substrate. We evaluated non-doped DLC and Ag-DLC coatings in a simulated bile model that mimics physiological conditions relevant to biliary drainage. Importantly, our approach builds upon the deposition parameters and antimicrobial insights of Harrasser et al. but extends their application to a different material and organ system. Accordingly, we formulated the following research questions: (i) Can DLC and Ag-DLC coatings be successfully applied to thermally sensitive PE stents using the employed DLC processes without damaging the substrate? (ii) Do these coatings reduce microbial adhesion and bile sludge accumulation under in vitro conditions mimicking the biliary tract environment?

We hypothesize that Ag-DLC coatings exert a dual anti-biofouling effect—reducing both microbial colonization and sludge accumulation—which may ultimately prolong stent patency and reduce the need for repeated interventions in the future.

## 2. Materials and Methods

### 2.1. Microorganisms

*E. faecalis* (ATCC^®^29212™), *E. coli* (ATCC^®^25922™), and *C. albicans* (ATCC^®^ 90028™) were selected as test species due to their prevalence in bile duct stents within the first 15 days post-insertion [3].

Microorganisms were pre-cultured on Columbia Blood Agar (CBA) (Becton Dickinson GmbH, Heidelberg, Germany) or Mueller–Hinton Agar (MHA) (Sigma-Aldrich, Merck KGaA, Darmstadt, Germany), and in Mueller–Hinton Broth II (MHB) (Becton Dickinson GmbH, Heidelberg, Germany) or Dulbecco’s Modified Eagle’s Medium (Sigma-Aldrich, Merck KGaA, Darmstadt, Germany) to prepare standardized bacterial suspensions for testing.

### 2.2. Biliary Plastic Stents

Uncoated PE biliary straight stents (10 French, EndoStay^®^, Peter Pflugbeil GmbH, Zorneding, Germany) served as reference standards. Stents were cut into 10.0 mm sections for testing, while 5.0 mm sections were prepared for scanning electron microscopy (SEM) in the bile incubation model.

### 2.3. Coating Solutions for Plastic Stents

DLC was investigated as a novel anti-adhesive coating for biliary plastic stents. DLC refers to a class of amorphous carbon thin-film materials with diamond-like properties [22]. AxynTeC Dünnschichttechnik GmbH, Augsburg, Germany, applied three coatings, namely DLC-1 (a-C:H), DLC-2 (ta-C), and DLC-2-Ag (ta-C:Ag). DLC-1, previously approved for coating metallic surgical instruments, was adapted for plastic stents using the PlasmaImpax process, a plasma-enhanced chemical vapor deposition (PECVD) method. The PlasmaImpax process is a hybrid technique combining plasma-assisted chemical vapor deposition (CVD) and ion implantation. This allows for the deposition of hydrogenated amorphous carbon (a-C:H) coatings at low substrate temperatures (approximately 200–250 °C), suitable for polymer substrates. The resulting coatings feature a layer thickness of 2–3 µm, a low coefficient of friction, and high hardness due to an sp^3^ bonding content of 30–50%, while maintaining elasticity [23]. DLC-2 was applied via the PLD method. PLD is a physical vapor deposition method in which a pulsed laser beam ablates a solid carbon target. The resulting plasma plume deposits a hydrogen-free ta-C film on the substrate. This method allows precise control of the sp^2^/sp^3^ ratio and coating characteristics. In the case of DLC-2-Ag, the film was additionally doped with Ag ions during the PLD process [24]. All stents—both plastic and DLC-coated—were sterilized in a drying oven at 80 °C prior to their use in the experimental series. Table 1 gives an overview of the different coating solutions and processes.

### 2.4. Quantification of Microbial Test Organisms

Microbial suspensions and supernatants were quantified using a spectrophotometer (Biophotometer, Eppendorf GmbH, Hamburg, Germany) by measuring optical density at 600 nm (OD 600) to produce standardized inocula. To determine pathogen adherence, stents were eluted, and serial dilutions were plated on agar to quantify colony-forming units (CFU). After incubation on CBA plates, colonies were imaged and counted using ImageJ 1.52a, Open Access Freeware software, to calculate CFU/mL concentrations.

### 2.5. Agar Diffusion Assay

The agar diffusion assay (zone-of-inhibition assay) is a well-established microbiological method for assessing the antimicrobial efficacy of drug-releasing samples. In this study, it was used to evaluate DLC-2-Ag stent samples.

**Figure 1:**
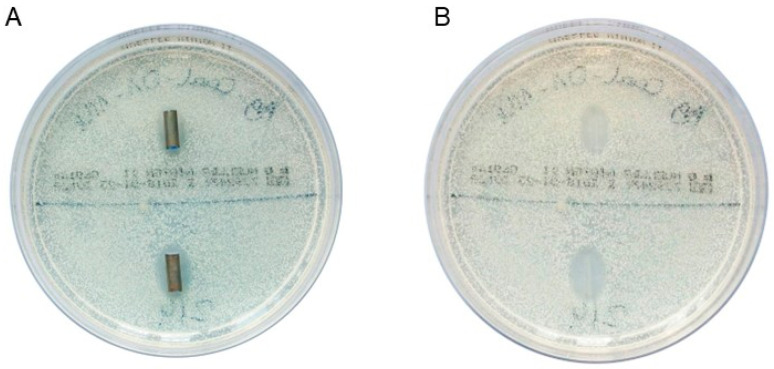
(**A**) DLC-2-Ag-coated stent samples after 1 day of incubation in a culture of *Candida albicans* on Mueller–Hinton agar. (**B**) Inhibition zones observed after removing the stent samples. Ag, silver; DLC, diamond-like carbon.

Liquid cultures of *E. faecalis*, *E. coli*, and *C. albicans* were prepared, adjusted to OD 600 of 0.1, and then 400 µL of each culture was plated on MHA agar. Once the liquid was absorbed, the stent samples were placed on the agar using sterile tweezers and incubated at 37 °C for 24 h. The following day, the stents were transferred to fresh MHA plates and examined for microbial growth inhibition. The inhibition zone diameter was measured using calipers. For every microorganism, four stent samples were investigated (*n* = 4). Figure 1A shows the two DLC-2-Ag-coated stent samples after 1 day of incubation in a culture of *C. albicans.* Figure 1B shows the inhibition zones after removing the stent samples.

### 2.6. Microbial Adhesion Assay (Using Monocultures in MHB for Stent Incubation)

A microbial adhesion assay was conducted to quantify viable microorganisms adhering to the stent surfaces. Stent samples were incubated in bacterial suspensions using monocultures in MHB, and adhered bacteria were detached via sonication. The number of CFU was determined by plating the bacterial suspension on CBA agar followed by 24 h incubation, to quantify adhered bacteria. Additionally, bacterial load in the supernatant was assessed for drug-releasing stents to evaluate the samples’ potential antimicrobial effects on the surrounding environment.

The adhesion of *E. faecalis* was tested on DLC-1 and DLC-2 samples, while *E. coli* adhesion was tested on DLC-2-Ag samples. Two test series were conducted for DLC-1 (*n* = 8 for both series), one for DLC-2 (*n* = 8), and three for DLC-2-Ag (*n* = 7 for all three series regarding the supernatant; *n* = 7 and *n* = 8 for test series 2 and 3 regarding surface adhesion), all incubated in a bacterial suspension without human bile (Supplementary Table S1).

The adhesion assay spanned 3 days per experimental round: Day 1: Preparation and incubation—stent samples were secured at the bottom of the wells in a microtiter plate (Falcon^®^ 24-well TC-treated plates (Corning Life Sciences, Tewksbury, MA, USA; distributed by VWR International GmbH, Darmstadt, Germany). Subsequently, stents were exposed to bacterial suspensions with adjusted initial densities and incubated at 37 °C for 24 h. Initial bacterial densities were as follows: DLC-1: 1 × 10^4^/mL (test series 1) and 1 × 10^3^/mL (test series 2); DLC-2: 1 × 10^4^/mL; DLC-2-Ag: 3 × 10^5^/mL (test series 1 and 2) and 3 × 10^4^/mL (test series 3). Day 2: Supernatant and surface analysis—bacterial suspensions from the supernatant were collected, chemically neutralized, diluted 10-fold, and plated for CFU counting. Stents were washed, sonicated, and vortexed to detach adhered bacteria, which were then plated using the same method. Day 3: colony counting—CFU were counted from CBA plates to quantify bacteria in both the supernatant and stent surfaces. This modified protocol builds on methods established by Heidenau et al. [25] and Obermeier et al. [26].

### 2.7. Bile Incubation Model (Using Co-Culture of Main Pathogens in Human Bile for Stent Incubation)

Stent samples (DLC-1, DLC-2, and DLC-2-Ag) were incubated in human bile inoculated with defined concentrations of 1 × 10^3^/mL of the test strain (*E. faecalis*, *E. coli*, and *C. albicans*) to simulate physiological conditions inside the bile duct. Human bile samples were collected during elective cholecystectomies at the TUM School of Medicine and Health, Department of Surgery, University Medical Center, Technical University of Munich, Germany. To ensure defined microbiological culture conditions, it was necessary to maintain sterility. Sterility was confirmed by incubating 100 µL of bile at 37 °C for 24 h on CBA plates. Only pooled human bile samples without colony formation were used in experiments. Additional microbiological analyses were conducted to investigate the non-sterile bile obtained from patients and to assess the pathogenic environment affecting stent materials in situ.

### 2.8. Collection and Characterization of Human Bile for in Vitro Testing

The aerobic bacterial spectra of non-sterile bile samples were analyzed using a matrix-assisted laser desorption/ionization time-of-flight (MALDI-TOF) mass spectrometer (TUM School of Medicine and Health, Institute for Medical Microbiology, Immunology and Hygiene, TUM University Hospital, Munich, Germany)

**Figure 2:**
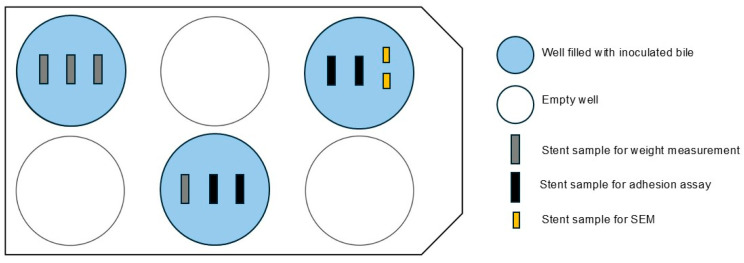
Arrangement of the titer plates in the bile incubation model. SEM, scanning electron microscopy.

For sterile bile samples, *E. faecalis*, *E. coli*, and *C. albicans* were added to achieve a total bacterial density of 3 × 10^3^/mL. Stent samples were secured with paraffin inside a microtiter plate (Falcon^®^ 6-well clear flat bottom TC-treated cell culture plates (Corning Life Sciences, Tewksbury, MA, USA), obtained via VWR International GmbH, Darmstadt, Germany), with separate plates for each stent type. Wells were filled with 7.5 mL of inoculated bile, sealed, and incubated at 37 °C on a shaker (Polymax 1040, Heidolph Scientific Products, Schwabach, Germany) at 2 revolutions per minute to simulate bile flow for 5 or 14 days. Figure 2 shows the arrangement of the multi-well plates in the bile incubation model.

Microbial adhesion to the stent surface and bacterial concentration in the surrounding bile were assessed using an adhesion assay. Stents were weighed pre- and post-incubation to quantify sludge accumulation following protocols by Dowidar et al. [27] and Farnbacher et al. [28]. Weighing was performed using a precision balance (ATL-224, Sartorius Acculab Atilon, Göttingen, Germany), and stents were stored in Stericlin^®^ bags. After incubation, stents were washed in phosphate-buffered saline, dried on mounted pipette tips to remove non-adherent sludge, and air-dried for 3 days to ensure a stable weight before final measurement.

High-resolution surface changes were analyzed using SEM. After incubation, stents were fixed with glutaraldehyde in phosphate-buffered saline, dehydrated using an ascending ethanol series, and dried with hexamethyldisilazane. Samples were examined using a ZEISS SIGMA VP Field Emission SEM (Carl Zeiss AG, Oberkochen, Germany) at the Institute of Water Chemistry, Technical University of Munich. Non-incubated native samples were also analyzed to compare the surface properties of PE- and DLC-coated stents.

The bile incubation model included three experimental rounds (Table 2). In round one (14 days), all three analytical methods (adhesion assay, weight analysis, and SEM) were applied. Round two (14 days) included the adhesion assay and weight analysis; SEM was omitted as a significant portion of the adherent sludge detached during preparation. The datasets from rounds one and two were statistically standardized, and results were based on both test series (adhesion assay: *n* = 12 for surface adhesion, *n* = 3 for the supernatant; weight analysis: *n* = 16). Round three (5 days) assessed the short-term anti-adhesive effect using an adhesion assay (*n* = 8 for surface adhesion, *n* = 2 for the supernatant).

### 2.9. Statistics

Statistical analyses were performed using Microsoft Excel, Version 16.92 (Microsoft Corporation, Redmond, WA, USA). The Kolmogorov–Smirnov test assessed normal distribution. Normally distributed datasets were analyzed using an independent-sample *t*-test, while non-normally distributed datasets were evaluated with the Mann–Whitney U test. All tests were two-sided, and statistical significance was set at *p* < 0.05.

### 2.10. Ethics

The use of human bile from patients undergoing elective cholecystectomy was approved by the Ethics Committee of the Technical University of Munich (approval number 391/18). This study adhered to the principles of the Declaration of Helsinki.

## 3. Results

### 3.1. Characteristics of Collected Human Bile

A total of 930.3 mL of human bile was collected from 65 patients, including 49 sterile samples (664.8 mL) and 15 non-sterile samples (263.5 mL). One sample dried during transport and was unusable. The average sample volume was 14.3 mL. Seven of the 15 non-sterile samples underwent MALDI-TOF analysis, identifying 8 pathogens. The most common were *E. faecalis* (*n* = 4) and *E. coli* (*n* = 2), while *C. albicans*, *Serratia marcescens*, *Hafnia alvei*, *Streptococcus gallolyticus*, *Citrobacter braakii*, and *Proteus hauseri* were also detected (*n* = 1). Table 3 shows an overview of the detected pathogens in non-sterile human bile.

### 3.2. Results of Microbial Anti-Adhesion Assay

#### 3.2.1. DLC-1 Coatings

More adherent bacteria were found on DLC-1 stents than on uncoated stents. The mean pathogen count on DLC-1 stents was 2.83 × 10^6^ colony forming units (CFU)/mL (standard deviation [SD] 3.96 × 10^6^ CFU/mL) in experimental round 1 and 1.15 × 10^7^ CFU/mL (SD 2.40 × 10^7^ CFU/mL) in round 2, while on uncoated stents, mean pathogen counts were 7.91 × 10^5^ CFU/mL (SD 8.39 × 10^5^ CFU/mL) in round 1 and 8.52 × 10^6^ CFU/mL (SD 7.59 × 10^6^ CFU/mL) in round 2. Bacterial growth on DLC-1 stents was, thus, 0.6 log-levels higher in round 1 (*p* = 0.19) and 0.1 log-levels higher in round 2 (*p* = 0.75) than on uncoated stents. The results of the growth of *E. faecalis* on DLC-1-coated stents are illustrated in Table 4.

**Figure 3:**
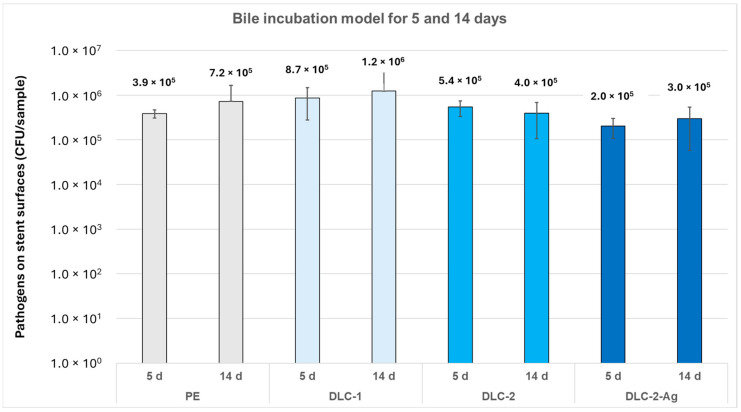
Pathogen growth on the surface of the stent samples after 5 and 14 days of incubation. Ag, silver; d, days; DLC, diamond-like carbon; PE, polyethylene.

After five days of incubation in pooled human bile inoculated with *E. faecalis*, *E. coli,* and *C. albicans*, DLC-1 stents had more adherent bacteria than uncoated stents, which is illustrated in Figure 3. The bacterial count on DLC-1 stents was 8.67 × 10^5^ CFU/mL (SD 5.92 × 10^5^ CFU/mL), 0.4 log-levels higher than on uncoated stents with 3.86 × 10^5^ CFU/mL (SD 8.16 × 10^4^ CFU/mL) (*p* = 0.06) (Supplementary Table S2). In the supernatant, bacterial growth was 0.3 log-levels lower with DLC-1 stents (4.46 × 10^6^ CFU/mL [SD 2.38 × 10^6^ CFU/mL]) than with uncoated stents (9.59 × 10^6^ CFU/mL [SD 1.54 × 10^6^ CFU/mL]) (*p* = 0.12) after 5 days (Supplementary Table S3).

After 14 days of incubation, bacterial growth on DLC-1 stents reached 1.24 × 10^6^ CFU/mL (SD 2.05 × 10^6^ CFU/mL), a 0.2 log-level increase compared to uncoated stents (7.24 × 10^5^ CFU/mL [SD 9.42 × 10^5^ CFU/mL]) (*p* = 0.44) (Supplementary Table S4). Figure 3 shows that after 14 days, most adherent bacteria were detected on DLC-1 stents.

In the supernatant, bacterial growth was 0.7 log-levels lower with DLC-1 stents (9.54 × 10^6^ CFU/mL [SD 1.00 × 10^7^ CFU/mL]) than with uncoated stents (4.40 × 10^7^ CFU/mL [SD 3.22 × 10^6^ CFU/mL]) (*p* = 0.13) after 14 days (Supplementary Table S5).

Additionally, after 14 days of incubation, DLC-1 stents exhibited less weight gain (9.1 mg, SD 17.1 mg) than uncoated stents (11.9 mg, SD 18.1 mg) (*p* > 0.05) (Table 5).

#### 3.2.2. DLC-2-Coatings

Like the DLC-1 stents, DLC-2-coated stents had more adherent bacteria than uncoated stents. DLC-2-coated stents contained 1.05 × 10^6^ CFU/mL (SD 1.33 × 10^6^ CFU/mL), while PE stents had 2.89 × 10^5^ CFU/mL (SD 1.81 × 10^5^ CFU/mL), a 0.6 log difference (*p* = 0.15), which is illustrated in Table 6.

After 5 days of incubation in human bile, bacterial growth was greater on DLC-2 stents than on uncoated stents but lower than that on DLC-1 stents (Figure 3). DLC-2 stents had 5.39 × 10^5^ CFU/mL of pathogens on their surface, 0.1 log higher than uncoated stents (*p* = 0.10) (Supplementary Table S2). As with DLC-1 stents, bacterial growth in the supernatant was less than that in the uncoated stents. The bacterial count in the supernatant of DLC-2 stents was 4.54 × 10^6^ CFU/mL (SD 3.21 × 10^6^ CFU/mL), 0.3 log lower than uncoated stents (*p* = 0.29) (Supplementary Table S3).

After incubation for 14 days, bacterial growth on DLC-2 stents was 3.98 × 10^5^ CFU/mL, thus it was 0.3 log lower than uncoated stents (*p* = 0.31) (Supplementary Table S4). In comparison, there was less bacterial growth on DLC2-2 stents than on DLC-1 stents (Figure 3). The microbial growth in the supernatant after 14 days was 0.2 log-levels lower with DLC-2 stents (2.71 × 10^7^ CFU/mL) compared to uncoated stents, but this difference was smaller than with DLC-1 stents (Supplementary Table S5). Due to the small sample size, no statistical test could be performed. DLC-2 stents showed the greatest weight gain, at 16.3 mg (SD 26.6 mg), corresponding to a 4.4 mg increase compared to uncoated stents (*p* > 0.05) (Table 5).

#### 3.2.3. DLC-2-Ag Coatings

As with DLC-1- and DLC-2-covered stents, more adherent bacteria were detected on DLC-2-Ag-coated stents than on uncoated PE stents. DLC-2-Ag stents had 3.35 × 10^4^ CFU/mL (SD 4.97 × 10^4^ CFU/mL) and 3.81 × 10^4^ CFU/mL (SD 8.03 × 10^4^ CFU/mL) *E. coli* adherent to their surface, compared to 7.68 × 10^3^ CFU/mL (SD 1.10 × 10^4^ CFU/mL) and 2.07 × 10^3^ CFU/mL (SD 2.70 × 10^3^ CFU/mL) on uncoated stents. Bacterial growth on DLC-2-Ag stents was 0.6 log-levels higher (*p* = 0.19) in round one and 1.3 log-levels higher (*p* = 0.28) in round two, the largest increase among the coatings tested. Table 7 shows the results of both experimental series.

In the bile incubation model with a 5-day period, DLC-2-Ag was the only coating to show reduced bacterial growth compared to DLC-1, DLC-2, and uncoated stents. DLC-2-Ag stents had 2.04 × 10^5^ CFU/mL (SD 9.59 × 10^4^ CFU/mL) of pathogens on their surface, a 0.3 log reduction compared to uncoated stents (*p* = 0.00) (Supplementary Table S2). In the supernatant, bacterial growth after 5 days (6.23 × 10^6^ CFU/mL [SD 2.45 × 10^6^ CFU/mL]) was 0.2 log-levels less compared to uncoated stents (*p* = 0.24), similar to DLC-1 and DLC-2 stents (Supplementary Table S3).

After 14 days, the DLC-2-Ag stents showed a reduction in bacterial growth, similar to that of the DLC-2 stents (Figure 3). The bacterial growth was 3.00 × 10^5^ CFU/mL (SD 2.42 × 10^5^ CFU/mL), a reduction of 0.4 log-levels compared to uncoated stents (*p* = 0.16) (Supplementary Table S4). Regarding the bacterial growth in the supernatant, the results are comparable to those after an incubation of 5 days: bacterial growth in DLC-2-Ag stents counted for 3.85 × 10^7^ CFU/mL (SD 2.99 × 10^7^ CFU/mL) and was 0.1 log-levels less than in uncoated stents (*p* = 0.78) (Supplementary Table S5). Compared to DLC-1 stents, DLC-2-Ag stents showed a smaller weight gain after 14 days (8.3 mg [SD 12.3 mg]) than uncoated stents (*p* > 0.05) (Table 5).

**Figure 4:**
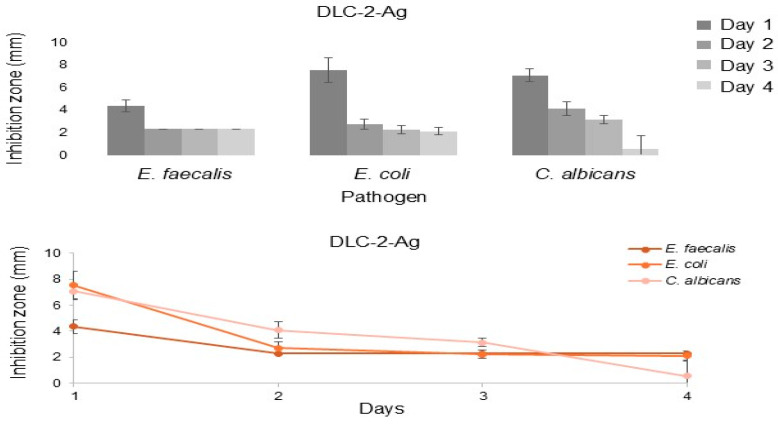
Inhibition zones formed by DLC-2-Ag-covered PE stents after 1, 2, 3, and 4 days. Ag, silver; *C. albicans*, *Candida albicans*; DLC, diamond-like carbon; *E. coli*, *Escherichia coli*; *E. faecalis*, *Enterococcus faecalis*; PE, polyethylene.

The antimicrobial efficacy of DLC-2-Ag-coated stents was assessed using an agar diffusion test. DLC-2-Ag-coated stents formed inhibition zones against *E. faecalis*, *E. coli,* and *C. albicans* over 4 days. Inhibition zones were the largest for *E. coli* (7.5 mm [SD 1.1 mm]) and *C. albicans* (7.1 mm [SD 0.6 mm]) on day one. By day four, the inhibition zones for *E. faecalis* (2.3 mm [SD 0.0 mm]) and *E. coli* (2.1 mm [SD 0.4 mm]) were similar, and that of *C. albicans* (0.6 mm [SD 1.2 mm]) was the smallest (Figure 4).

#### 3.2.4. SEM

**Figure 5:**
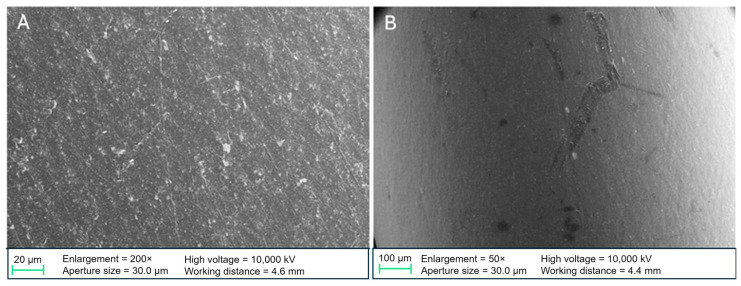
(**A**) Polyethylene stent with DLC-2 coating, non-incubated. (**B**) Polyethylene stent with DLC-2-Ag coating, non-incubated. Ag, silver; DLC, diamond-like carbon.

**Figure 6:**
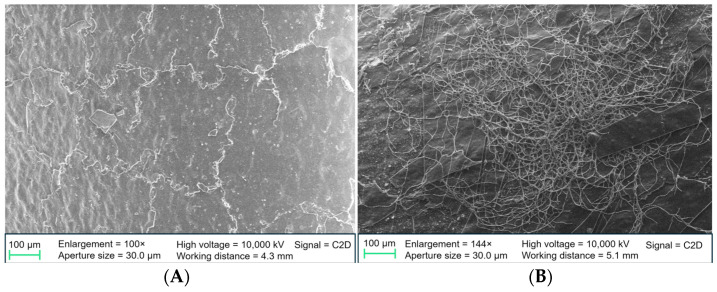
(**A**) Polyethylene stent with DLC-1 coating, non-incubated. (**B**) Pathogen material (likely *Candida albicans*) on a DLC-1-coated stent. DLC, diamond-like carbon.

**Figure 7:**
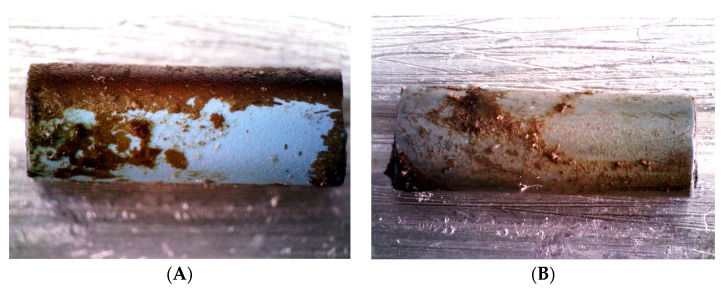
Polyethylene stents with DLC coatings after 14 days of incubation in inoculated human bile ((**A**) DLC-1 and (**B**) DLC-2-Ag). Ag, silver; DLC, diamond-like carbon.

SEM images were captured of non-incubated and 14-day-incubated stent samples. Images of non-incubated stents showed cracks and exfoliation of the coating, suggesting an irregular coating process. These coating deficiencies were visible in all the examined DLC modifications (Figure 5A,B and Figure 6A). Few microorganisms were visualized on the incubated stent samples, as most of the sludge was detached during SEM preparation. Most microorganisms were detectable on DLC-1 stents (Figure 6B). Furthermore, the coatings did not show sufficient adhesion after a longer period of exposure to inoculated human bile. Figure 7 illustrates that most DLC-1 (Figure 7A) and DLC-2-Ag (Figure 7B) coatings detached after 14 days in human bile, and the same applies to DLC-2 coatings as well.

## 4. Discussion

Occlusion of biliary PE stents is a common complication following ERC due to sludge accumulation and biofilm formation from bacterial adhesion [3]. DLC, a polymorphic group of carbon compounds, has anti-adhesive properties, making it suitable as a coating [29]. Doping with Ag ions further enhances antimicrobial effectiveness [30].

This study is the first to systematically investigate the anti-adhesive effects of DLC coatings with Ag doping on PE biliary stents. The stent material was incubated in human bile with a defined bacterial density, simulating the clinical situation in vitro, a novel approach.

*E. faecalis* and *E. coli* were the most commonly detected pathogens in non-sterile human bile from patients who underwent elective cholecystectomy, consistent with previous studies on bile pathogens [2,3], supporting the choice of *E. faecalis*, *E. coli*, and *C. albicans* for testing. Neither DLC-1- nor DLC-2-coated PE stents exhibited anti-adhesive effects against *E. coli* or *E. faecalis*. Kulikovsky et al. [31] conducted an in vitro study with plastic biliary stents coated with DLC-Ag incubated with human bile. Agar diffusion tests showed inhibition zones around DLC-Ag-coated stents, unlike the reference samples. However, this study did not analyze stents coated exclusively with DLC (without Ag doping) and had methodological deficiencies.

Numerous plastic stents and catheters, especially in urology, have been developed to maintain urine flow [32]. Two comparable studies examined the effects of DLC coatings on urethral stents and bladder catheters. Watari et al. [33] coated the inner lumen of a silicon tube (2 mm diameter) using alternating-current (AC) high-voltage methane PECVD. They found reduced adherence of *Pseudomonas aeruginosa* (*P. aeruginosa*) and *E. coli* compared with uncoated tubes and inhibition of *P. aeruginosa* biofilm and microcolony formation. Laube et al. [34] showed similar results in vivo with DLC-coated polyurethane ureteral stents. They also used the PECVD method, but the comparability of our study is limited due to the unreported coating quality and possible biofilm assessment by cystoscopy alone.

Differences between our results and those of Watari et al. may be due to variations in coating quality. Our SEM images revealed delamination and cracks in the DLC coating, particularly after fluid incubation, leading to increased microbial adhesion in these areas. The edges of the coating may serve as micro-scale niches where bacterial colonization is more likely than on smooth surfaces. This issue could stem from the cylindrical shape of the stents, which makes it difficult to apply DLC coatings uniformly. Excessive layer thickness could also contribute to delamination, though the exact thickness was not measured. However, standardized coatings from AxynTeC Dünnschichttechnik GmbH, the company responsible for the coatings, typically range from 1–6 µm [35].

While DLC coatings for metallic substrates have been optimized, adapting these techniques to polymeric silicone remains challenging. DLC adhesion depends on such factors as substrate lattice spacing and coating temperature. Lower coating temperatures, pretreatments, and intermediate layers are essential to ensure strong adhesion to polymeric silicone [36]. Future research should focus on refining the coating process to enhance adhesion and quality, potentially by adjusting parameters during production (e.g., variations in sp^2^ and sp^3^ bonding) or using intermediate layers as adhesion promoters. CVD and PECVD coatings have shown significant promise in enhancing mechanical durability and reducing microbial adhesion in preclinical and in vitro studies, but their clinical effectiveness—specifically in biliary drainage stents—remains experimental. To our knowledge, no large-scale clinical trials have definitively confirmed improved clinical outcomes solely due to CVD/PECVD-applied DLC coatings in the biliary setting. Nevertheless, CVD coatings have demonstrated benefits in related medical applications [12,37]. These findings support the rationale for applying similar technologies to polymeric biliary stents and the need for further in vivo and clinical research.

Another future research objective is to compare the established PECVD technique with the more advanced AC high-voltage methane plasma CVD method. Watari et al. reported a high-quality DLC coating with a smooth surface and structural integrity even after 2 months of urine exposure, achieved using AC high-voltage methane plasma CVD. This technique is specifically tailored for intraluminal coatings of small-diameter stents, as methane plasma remains confined within the tube, enabling uniform deposition [38]. However, due to its complexity and limited availability, this method was not used in our study. A key limitation of our approach is that PE stents could only be coated externally due to their small diameter and the coating process itself. This is significant because bacterial biofilms tend to develop inside stents rather than on their outer surface. PECVD, while widely used for coating planar and metallic objects, poses challenges when applied to the curved, narrow lumen of bile duct stents due to restricted accessibility [39]. Further studies should validate the use of AC high-voltage methane plasma CVD for intraluminal coatings, as it represents a promising advancement in addressing one of the primary limitations of current stent-coating methods.

DLC-2-Ag formed an inhibition zone in agar diffusion assays against *E. coli*, *E. faecalis*, and *C. albicans* for 4 days. The antibacterial action of Ag involves binding to cellular membranes, enzymes, and nucleic acids, disrupting the respiratory chain, and impairing aerobic metabolism in microorganisms [30]. Ag nanoparticles (AgNPs) can also adhere to bacterial cell walls, increasing membrane permeability and causing disintegration [21]. Several in vitro studies have demonstrated Ag’s antimicrobial effects. Yang et al. [21] showed that doping plastic biliary stents with Ag prolonged patency and provided broad antimicrobial efficacy against *E. coli*, *Staphylococcus aureus*, *Enterococcus*, and *P. aeruginosa*. Wen et al. [40] found that polyurethane stents doped with AgNPs reduced bacterial adhesion when preclinically implanted in pigs with bacterial cholangitis. Similar findings were reported by Park et al. [41], Lee et al. [42], and Yamabe et al. [43] in studies on Ag-doped metal and plastic biliary stents, reinforcing Ag’s antimicrobial ability. Metal stents inherently have longer patency than plastic stents due to their larger diameter, whereas plastic biliary stents are typically replaced every 3 months [7]. Although Ag doping improved plastic stent patency in non-clinical studies, its duration remains debatable, given the small stent diameter and limited Ag release period [30]. In this study, DLC-2-Ag-coated stents reduced bacterial adhesion in the bile incubation model for over 5 days but had no relevant effect after 14 days. In contrast, Yang et al. reported sustained antimicrobial efficacy of AgNPs doping for up to 48 weeks due to the slow release of Ag^+^ in biliary stents. Ag’s antimicrobial effects appear proportional to its concentration and dependent on release dynamics [16]. This release can be modulated by incorporating additional carrier elements or compounds, enhancing Ag’s mechanical stability [44]. In addition to carbon, silicon polymers and titanium are suitable Ag carriers for inhibiting bacterial growth on surfaces [17,18,42,45]. In our study, a single predefined Ag concentration was used, based on previous data indicating sufficient antimicrobial activity without cytotoxicity. However, systematic variation of the Ag dopant concentration was not performed and will be the subject of future investigations. Dose-dependent efficacy and release profiles will be essential to optimize long-term stent patency under biliary conditions. In addition to silver, alternative antimicrobial ions, such as copper (Cu) and zinc (Zn), are also under investigation for surface modification of biomedical devices. Previous studies have shown that Cu-doped DLC coatings can reduce bacterial growth depending on the deposition method, although their performance in vivo may be limited by increased encrustation in fluid-exposed environments [17,46]. Zn has demonstrated strong in vitro antimicrobial activity and biocompatibility in implant settings [47]. Alternative dopants may complement silver’s antimicrobial effects and should be systematically evaluated in future studies under biliary-specific conditions, considering ion release kinetics, biocompatibility, and biofilm inhibition potential. Therefore, the novel coated stents in this study require further optimization, with Ag release characterized and adapted for clinical bile duct applications to maintain patency for at least 3 months.

In summary, this is, to our knowledge, the first study to transfer DLC-Ag coating techniques to thermosensitive polymeric biliary stents and to evaluate their anti-adhesive performance under simulated bile conditions. Unlike previous works performed on HDPE or metallic substrates, our study mimics the physiological complexity of the biliary tract, including bile composition and relevant microbial species. These findings suggest a potential translational benefit for improving the patency and longevity of biliary drainage systems.

Our results demonstrate that both DLC and DLC-Ag coatings can be applied to thermosensitive PE stents using a low-temperature PECVD-based process without compromising the substrate’s integrity, thereby affirmatively addressing research question (i). Furthermore, quantitative adhesion tests and SEM analyses revealed that DLC-Ag coatings significantly reduced microbial attachment and bile sludge accumulation compared to uncoated controls. While variability remained high and some comparisons did not reach statistical significance, the observed trends support the hypothesis formulated in question (ii) and indicate a promising dual anti-biofouling effect.

Certain limitations must be addressed. This study was conducted in an in vitro setting with standardized human bile and selected microbial species. While this model approximates physiological conditions, in vivo factors, such as bile flow dynamics, immune interactions, and long-term biocompatibility, require further investigation. Future studies should include animal models or clinical pilot trials to evaluate the translational applicability of DLC-Ag-coated biliary stents.

## Figures and Tables

**Table 1:** Overview of different DLC coating solutions and coating processes.

Coating	DLC-1	DLC-2	DLC-2-Ag
DLC modification	a-C:H	ta-C	ta-C:Ag
Coating process	PlasmaImpax (P3I)	PLD	PLD

a, amorphous; Ag, silver; C, carbon; DLC, diamond-like carbon; H, hydrogenated carbon; PLD, pulsed laser deposition; ta, tetrahedral amorphous.

**Table 2:** Overview of the experimental series of the bile incubation model.

	Experimental Round 1		Experimental Round 2		Experimental Round 3	
Duration	14 days				5 days	
Samples	PE, DLC-1, DLC-2, DLC-2-Ag					
Methods	Adhesion assay:		Adhesion assay:		Adhesion assay	
	Supernatant	*n* = 2	Supernatant	*n* = 1	Supernatant	*n* = 2
	Surface adhesion	*n* = 4	Surface adhesion	*n* = 8	Surface adhesion	*n* = 8
	Weight analysis	*n* = 4	Weight analysis	*n* = 12		
	SEM	*n* = 2				

Ag, silver; DLC, diamond-like carbon; *n*, total number; PE, polyethylene; SEM, scanning electron microscopy.

**Table 3:** Pathogen spectrum of non-sterile human bile.

Detected Pathogens in Non-Sterile Human Bile	Total Number
*Enterococcus faecalis*	4
*Escherichia coli*	2
*Candida albicans*	1
*Serratia marcescens*	1
*Hafnia alvei*	1
*Streptococcus gallolyticus*	1
*Citrobacter braakii*	1
*Proteus hauseri*	1

**Table 4:** Statistical analysis of the growth of *E. faecalis* on polyethylene and DLC-1-coated stents.

Experimental Round	Round 1 (*n* = 8)		Round 2 (*n* = 8)	
Stent sample	PE	DLC-1	PE	DLC-1
CFU/mL (mean)	7.91 × 10^5^	2.83 × 10^6^	8.52 × 10^6^	1.15 × 10^7^
Pathogen count/mL (SD)	8.39 × 10^5^	3.96 × 10^6^	7.59 × 10^6^	2.40 × 10^7^
Differences in log levels	0.6		0.1	
*p*-value (*t*-test)	*p* = 0.19		*p* = 0.75	
Statistical significance	n.s.		n.s.	

CFU, colony forming units; DLC, diamond-like carbon; *n*, total number; n.s., not significant; PE, polyethylene; SD, standard deviation.

**Table 5:** Weight measurements of uncoated polyethylene stents and stents with different coatings after 14 days of incubation in human bile.

Stent Sample	PE	DLC-1	DLC-2	DLC-2-Ag
Total number	*n* = 16	*n* = 16	*n* = 13	*n* = 16
Weight gain, mg (mean)	11.9	9.1	16.3	8.3
Weight gain, mg (SD)	18.1	17.1	26.6	12.3
Difference in weight to PE, mg		−2.8	4.4	−3.7
*p*-value (Mann–Whitney U test)		*p* > 0.05	*p* > 0.05	*p* > 0.05

Ag, silver; DLC, diamond-like carbon; *n*, total number; PE, polyethylene; SD, standard deviation.

**Table 6:** Statistical analysis of the growth of *E. faecalis* on polyethylene and DLC-2-coated stents.

Experimental Round 1 (*n* = 8)		
Coating solution	PE	DLC-2
CFU/mL (mean)	2.89 × 10^5^	1.05 × 10^6^
CFU/mL (SD)	1.81 × 10^5^	1.33 × 10^6^
Differences in log levels	0.6	
*p*-value (*t*-test)	*p* = 0.15	

CFU, colony forming units; DLC, diamond-like carbon; *E. faecalis*, *Enterococcus faecalis*; *n*, total number; PE, polyethylene; SD, standard deviation.

**Table 7:** Statistical analysis of growth of *E. coli* on polyethylene and DLC-2-Ag-coated stents.

Experimental Round	Round 2 (*n* = 8)		Round 3 (*n* = 8)	
Coating solution	PE	DLC-2-Ag	PE	DLC-2-Ag
CFU/mL (mean)	7.68 × 10^3^	3.35 × 10^4^	2.07 × 10^3^	3.81 × 10^4^
CFU/mL (SD)	1.10 × 10^4^	4.97 × 10^4^	2.70 × 10^3^	8.03 × 10^4^
Differences in log levels	0.6		1.3	
*p*-value (*t*-test)	*p* = 0.19		*p* = 0.28	

Ag, silver; CFU, colony forming units; DLC, diamond-like carbon; *E. coli*, *Escherichia coli*; *n*, total number; PE, polyethylene; SD, standard deviation.

## Data Availability

The original contributions presented in this study are included in the article/Supplementary Material. Further inquiries can be directed to the corresponding author(s).

## Supplementary Materials

The following supporting information can be downloaded at: https://www.mdpi.com/article/10.3390/biomedicines13081950/s1. Supplementary Table S1: Overview of the experimental series of DLC-1-, DLC-2-, and DLC-2-Ag-coated stents. Supplementary Table S2: Statistical analysis of pathogen growth on the surface of stent samples after 5 days of incubation in human bile. Supplementary Table S3: Statistical analysis of pathogen growth in the supernatant of stent samples after 5 days of incubation in human bile. Supplementary Table S4: Statistical analysis of pathogen growth on the surface of stent samples after 14 days of incubation in human bile. Supplementary Table S5: Statistical analysis of pathogen growth in the supernatant of stent samples after 14 days of incubation in human bile.

## Abbreviations

The following abbreviations are used in this manuscript:

AC	Alternating current
Ag	Silver
AgNPs	Ag nanoparticles
CBA	Columbia Blood Agar
CFU	Colony-forming units
CVD	Chemical vapor deposition
DLC	Diamond-like carbon
DLC-1	Diamond-like carbon 1 (a-C:H)
DLC-2	Diamond-like carbon 2 (ta-C)
DLC-2-Ag	Diamond-like carbon with silver doping (ta-C:Ag)
ERC	Endoscopic retrograde cholangiography
HDPE	High-density polyethylene
MALDI-TOF	Matrix-assisted laser desorption/ionization time-of-flight
MHA	Mueller–Hinton Agar
MHB	Mueller–Hinton Broth
OD 600	Optical density at 600 nm
PE	Polyethylene
PECVD	Plasma-enhanced chemical vapor deposition
PLD	Pulsed laser deposition
SD	Standard deviation
SEM	Scanning electron microscopy
spp.	Species
