# Supplementary material for: Novel Coating Approaches for Polyethylene Biliary Stents to Reduce Microbial Adhesion, Prevent Biofilm Formation, and Prolong Stent Patency
Source: Biomedicines. 2025 Aug 9;13(8):1950. doi: 10.3390/biomedicines13081950 (PMC12383971; doi:10.3390/biomedicines13081950)
Supplement: Supplementary file 1 [file biomedicines-13-01950-s001.zip › biomedicines-3716302-supplementary.pdf]

Supplements

Supplementary Table S1. Overview of the e×perimental series of DLC-1, DLC-2, and DLC-2-Ag coated stents

**DLC-1**					
**E×perimental round**	**Round 1**		**Round 2**		
Type of test	Adhesion assay (n = 8)		Adhesion assay (n = 8)		
Strain	*E. faecalis*				
Initial concentration (CFU/ml)	1×10^4		1×10^3		
**DLC-2**					
**E×perimental round**	**Round 1**				
Type of test	Adhesion assay (n = 8)				
Strain	*E. faecalis*				
Initial concentration (CFU/ml)	1×10^4				
**DLC-2-Ag**					
**E×perimental round**	**Round 1**	**Round 2**		**Round 3**	
Type of test	Supernatant (n = 7)	Supernatant (n = 7)	Adhesion assay (n = 8)	Supernatant (n = 7)	Adhesion assay (n = 7)
Strain	*E. coli*				
Initial concentration (CFU/ml)	3×10^5	3×10^5		3×10^4	

Ag, silver; DLC, diamond-like carbon; *E. coli, Escherichia coli; E. faecalis, Enterococcus faecalis*; *n*, total number.

Supplementary Table S2. Statistical analysis of pathogen growth on the surface of stent samples after 5 days of incubation in human bile.

**Stent sample**	**PE**	**DLC-1**	**DLC-2**	**DLC-2-Ag**
Total number	n = 8	n = 8	n = 7	n = 8

CFU/ml, mean	3.86×10^5	8.67×10^5	5.39×10^5	2.04×10^5
CFU/ml, SD	8.16×10^4	5.92×10^5	2.06×10^5	9.59×10^4
Difference in log levels		0.4	0.1	-0.3
p-value (t-test)		p = 0.06	p = 0.10	p = 0.00

Ag, silver; CFU, colony forming units; DLC, diamond-like carbon; *n*, total number; PE, polyethylene; SD, standard deviation.

Supplementary Table S3. Statistical analysis of pathogen growth in the supernatant of stent samples after 5 days of incubation in human bile.

Stent sample	PE	DLC-1	DLC-2	DLC-2-Ag
Total number	*n* = 2	*n* = 2	*n* = 2	*n* = 2
CFU/ml, mean	9.59×10^6	4.46×10^6	4.54×10^6	6.23×10^6
CFU/ml, SD	1.54×10^6	2.38×10^6	3.21×10^6	2.45×10^6
Difference in log levels		-0.3	-0,3	-0.2
p-value (t-test)		p = 0.12	p = 0.29	p = 0.24

Ag, silver; CFU, colony forming units; DLC, diamond-like carbon; *n*, total number; PE, polyethylene; SD, standard deviation.

Supplementary Table S4. Statistical analysis of pathogen growth on the surface of stent samples after 14 days of incubation in human bile.

Stent sample	PE	DLC-1	DLC-2	DLC-2-Ag
Total number	*n* = 12	*n* = 12	*n* = 4	*n* = 12
CFU/ml, mean	7.24×10^5	1.24×10^6	3.98×10^5	3.00×10^5
CFU/ml, SD	9.42×10^5	2.05×10^6	2.92×10^5	2.42×10^5
Difference in log levels		0.2	-0.3	-0.4
p-value (t-test)		p = 0.44	p = 0.31	p = 0.16

Ag, silver; CFU, colony forming units; DLC, diamond-like carbon; *n*, total number; PE, polyethylene; SD, standard deviation.

Supplementary Table S5. Statistical analysis of pathogen growth in the supernatant of stent samples after 14 days of incubation in human bile.

**Stent sample**	**PE**	**DLC-1**	**DLC-2**	**DLC-2-Ag**
Total number	$n = 3$	$n = 2$	$n = 1$	$n = 3$
CFU/ml, mean	4.40×10^7	9.54×10^6	2.71×10^7	3.85×10^7
CFU/ml, SD	3.22×10^6	1.00×10^7	/	2.99×10^7
Difference in log levels		-0.7	-0,2	-0.1
p-value (t-test)		p = 0.13	/	p = 0.78

Ag, silver; CFU, colony forming units; DLC, diamond-like carbon; E, international units; *n*, total number; PE, polyethylene; SD, standard deviation.
